# Identification of genes differentially expressed during larval molting and metamorphosis of *Helicoverpa armigera*

**DOI:** 10.1186/1471-213X-7-73

**Published:** 2007-06-25

**Authors:** Du-Juan Dong, Hong-Juan He, Lian-Qin Chai, Xiao-Juan Jiang, Jin-Xing Wang, Xiao-Fan Zhao

**Affiliations:** 1 School of Life Sciences, Shandong University, Jinan 250100, China

## Abstract

**Background:**

Larval molting and metamorphosis are important physiological processes in the life cycle of the holometabolous insect. We used suppression subtractive hybridization (SSH) to identify genes differentially expressed during larval molting and metamorphosis.

**Results:**

We performed SSH between tissues from a variety of developmental stages, including molting 5th and feeding 6th instar larvae, metamorphically committed and feeding 5th instar larvae, and feeding 5th instar and metamorphically committed larvae. One hundred expressed sequence tags (ESTs) were identified and included 73 putative genes with similarity to known genes, and 27 unknown ESTs. SSH results were further characterized by dot blot, Northern blot, and RT-PCR. The expression levels of eleven genes were found to change during larval molting or metamorphosis, suggesting a functional role during these processes.

**Conclusion:**

These results provide a new set of genes expressed specifically during larval molt or metamorphosis that are candidates for further studies into the regulatory mechanisms of those stage-specific genes during larval molt and metamorphosis

## Background

Molting is a physiological process common to all ecdysozoan animals, including nematodes and arthropods, during which the old exoskeleton, or cuticle, is shed and replaced by a new exoskeleton. The life cycle of the holometabolous insect is characterized by a series of molts: larval molts, during which the larva progresses from one instar to the next, and metamorphic molts, which lead to pupation and eclosion. Generally, molting consists of two phases, the lethargus, or inactive phase, and ecdysis, or shedding of old cuticle. A cascade of physiological processes occurs during molting, including separation of the old cuticle from the underlying epidermis (apolysis), secretion of a new cuticle beneath the old, and finally shedding of the old exoskeleton [1]. Subsequent metamorphosis, the transformation of larva to pupa to adult, includes metamorphic molting. During this process, more complicated physiological processes occur, including histolysis of larval tissues, remodeling and formation of adult tissues, in addition to a molting cascade similar to the larval molt. Apoptotic and autophagic programmed cell death pathways are involved in tissue histolysis and remodeling during metamorphosis [2,3]. Insect larval molting and metamorphosis are governed by ecdysteroids (20-hydroxyecdysone, 20E) and juvenile hormone (JH), with 20E orchestrating the molting process and JH determining the nature of the molt [4]. In the presence of JH, 20E directs larval molting. Otherwise, 20E directs metamorphosis. Twenty hydroxyecdysone (20E) and JH levels increase during the late stages of the final (wandering) instar in *Manduca *larvae, before pupal ecdysis, and then decrease at the pupal ecdysis. 20E levels peak midway through the pupal stage, but JH does not [4]. Increasing evidence indicates that other hormones and receptors may contribute to the complex developmental pathways associated with metamorphosis [5]. However, the endocrine circuits that regulate molting in response to environmental and physiologic cues are not well understood. Moreover, little is known about the molecular mechanisms regulating release and *de novo *production of the new exoskeleton [6]. The molecular mechanisms underlying metamorphosis are also enigmatic, although some key regulatory genes have been identified, such as *Broad complex*, *E74B *and *E93 *[2]. In addition, very few genes downstream of Broad complex, E74B and E93 are identified. Therefore, the molecular mechanisms that lead to larval molt and metamorphosis are poorly understood. Nevertheless, much remains to be learned regarding the molecular regulatory processes governing larval molting and metamorphosis.

Tremendous efforts have been made to identify larval molting and metamorphosis genes. Many genes have been shown to be involved in molting or metamorphosis, such as the transcription factors *EcR*, *USP*, *HR3*, *Broad C *[4], and the programmed cell death pathway genes [7]. The powerful technique of DNA microarray has extended the single-gene approach to the genome level by allowing simultaneous comparison of transcript levels of thousands of genes [8]. Several microarray and functional genomic studies have led to the identification of genes that exhibit a change in expression level at the onset of metamorphosis. For example, microarray technology was used to investigate gene expression changes in response to 20E, and dependent on *EcR*, at the onset of metamorphosis in *Drosophila *[9]. Tissue-specific gene expression and ecdysone-regulated genomic networks have also been examined by microarray in *Drosophila *[10]. Another study found that about one-third of all *Drosophila *genes undergo changes in expression by examining whole insects at different developmental timepoints [11].

To date, genome-wide studies of molting and metamorphosis have been limited to *Drosophila *or *C. elegans *because of the availability of genomic sequence and commercial microarrays for these two models. Thus, no large-scale investigation of larval molting and metamorphosis genes has been reported for a non-Dipteran insect. The lepidopteran, *Helicoverpa armigera *is a notorious crop pest, the world over, and shares molting and metamorphosis features with *Manduca sexta *and *Bombyx mori*. Because no genomic sequence or commercial microarrays exist for *H. armigera*, large scale screening of genes expressed differentially expressed during molting and metamorphosis is a great challenge. Initial efforts to study the molecular regulation of these important life cycle changes focused on the *H. armigera *hormone receptor 3 (HHR3) and its expression patterns during larval molting [12]. To discover new larval molting genes in a higher throughput manner, we performed two-dimensional electrophoresis and identified 30 new proteins whose expression increased during larval molting [13]. However, many more genes involved in larval molting and metamorphosis in *H. armigera *remain to be identified.

Suppression subtractive hybridization (SSH) is a powerful method for identifying differentially expressed genes. In this technique, cDNA from one population of cells/tissues is used as the "tester," to reveal cDNAs unique to a second population, the "driver". Nucleotide adaptors are first added to tester cDNAs and these are then hybridized with an excess of driver cDNAs. cDNAs unique to the driver population can then be amplified by PCR, while adaptors from the tester pool suppress amplification of cDNAs common to tester and driver populations [14,15]. Here, we use SSH to identify genes from *H. armigera *that are differentially expressed during larval molting or metamorphosis. These include regulators, hydrolases, and other genes that will further the study of the regulatory mechanisms of insect larval molting and metamorphosis.

## Results

### Identification of genes differentially expressed during larval molting

**Figure 1:**
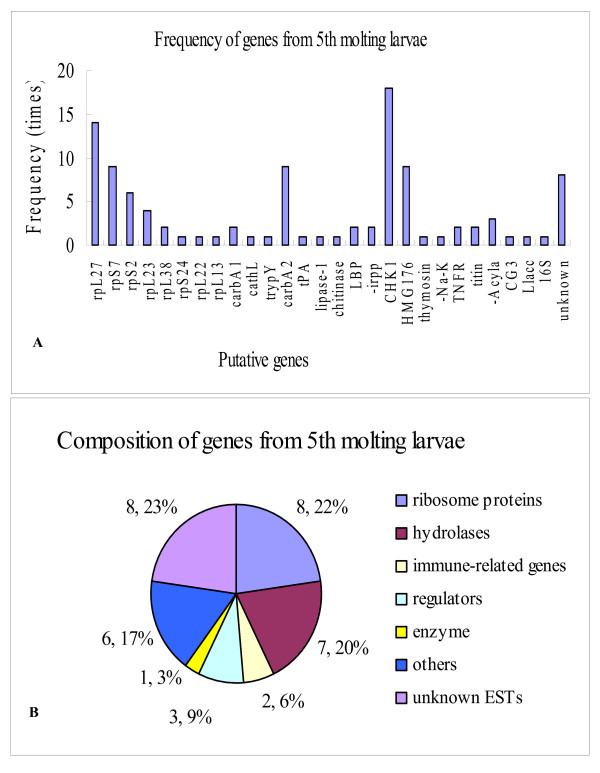
Genes identified in 5th instar molting larvae. A) Gene frequency. B) Gene transcription composition (number, percentage).

cDNAs from five separate tissues from molting 5th instar larvae (5th-HCS, with head capsule slippage, HCS) were used as the tester and cDNAs from feeding 6th instar larvae (6th-48 h) were used as the driver in five separate rounds of SSH, resulting in 200 cDNAs with molecular masses greater than 300–500 bp. Sequencing revealed 27 putative genes with similarity to annotated genes in Genbank by BLAST search, and 8 novel ESTs (Table 1). Very high frequency genes included the ribosome proteins genes *rpL27*, *S7*, *S2 *and *L23*. Other high frequency genes were *CHK1 checkpoint homolog*, *carboxypeptidase A2 *(*carbA2*) and *hmg176*. However, most genes appeared at a lower frequency of 1 or 2 times (Fig. 1A).

**Table 1:** Identification of putative genes from SSH of tester cDNA from molting 5th instar larvae and driver cDNA from feeding 6th instar larvae

**Classification**	**No**	**Tissue Sources **	**Similar genes in GenBank**	**E-value**	**Functions of the genes **
Ribosome proteins	1	head midgut epidermis	ribosomal protein L27 [*S. frugiperda*]	1e-55	participates in both 50 S subunit assembly and the peptidyl transferase reaction
	2	head	ribosomal protein S7 [*S.frugiperda*]	7e-23	initiating assembly of the head of the 30S subunit
	3	fat body midgut	ribosomal protein S2 [*B. mori*]	2e-40	maintaining balance between the small and large ribosomal subunits
	4	midgut	ribosomal protein L17/23 [*Apis mellifera*]	8e-17	regulator of the p53-MDM2 feedback regulation
	5	midgut	ribosomal protein L38 [*Plutella xylostella*]	2e-17	protein of 60 S ribosomal subunit
	6	haemocyte	ribosomal protein S24 [*B. mori*]	1e-21	protein of 40 S ribosomal subunit
	7	head	ribosomal protein L22 [*S. frugiperda*]	2e-29	large ribosomal subunit
	8	midgut	ribosomal protein L13 [*S. frugiperda*]	3e-43	60 S ribosomal subunit protein
Hydrolases	9	fat body	molting fluid carboxypeptidase A [*B. mori*]-1	3e-31	pupal ecdysis, recycling of the amino acids
	10	haemocyte	cathepsin L like protease [Glossina morsitans morsitans]	1e-25	larval molting and cuticle and eggshell remodeling
	11	midgut	trypsinogen Y precursor [*Gadus morhua*]	2e-06	hydrolase
	12	epidermis	molting fluid carboxypeptidase A [*B. mori*]-2	1e-54	molting-related
	13	epidermis	plasminogen activator, tissue [*Mus musculus*]	7e-07	hydrolase
	14	midgut	lipase-1 [*B. mandarina*]	2e-23	lipid digestion
	15	fatbody	chitinase [*H. armigera*] (BLAST by nucleotide acids)	0.42	hydrolase
Immune-related Genes	16	haemocyte	lipopolysaccharide binding protein [*B. mori*]	6e-26	immune
	17	fat body	immune reactive putative protease inhibitor PrInh6 [*Glossinamorsitans morsitans*]	2e-09	immune
Regulators	18	fat body haemocyte	CHK1 checkpoint homolog [*Xenopus tropicalis*]	2e-07	DNA damage and replication checkpoints
	19	midgut	CG13323-PA [*D. melanogaster*] (similar to hmg176)	6e-05	^-^
	20	epidermis	thymosin beta homologue [*Ciona intestinalis*]	0.55	actin-remodeling, wound healing, angiogenesis
enzyme	21	head	Na-K-ATPase [*Anopheles gambiae*]	2e-09	enzyme
Others	22	midgut	TNF receptor homolog 3 [*Rattus norvegicus*]	3	cellular proliferation, differentiation and programmed cell death
	23	epidermis	Titin-like protein [*B. mori*]	8e-15	roles in chromosomes and muscles
	24	midgut	Acyla-CoA binding protein [*B. mori*]	4e-06	intracellular acyl-CoA transporters
	25	fat body	hypothetical protein Llacc01000286 [*Lactococcus lactis subsp.cremoris *SK11]	1e-24	-
	26	head	CG32972-PB, isoform B [*D.melanogaster*]	0.011	-
	27	fat body	16S rRNA [*S. frugiperda*] (BLAST by nucleotide acids)	7e-17	-
Unknown ESTs	28–35	epidermis	Unknown proteins		-

Next, we grouped our genes according to predicted function, based on similarity to known genes in the National Center of Bioinformatics (NCBI) database, to produce seven main groups: ribosome proteins, rp), hydrolases, immune-related genes, regulators, enzyme, other genes, and unknown ESTs. Among those genes, eight encoded ribosome proteins (22% of the genes), and seven encoded hydrolases, including *carbA1 *and c *arbA2*,*cathepsin L *(*cathL*), *trypsinogen Y*, *tissue plasminogen activator *(*tPA*), *lipase*, and a *chitinase*-like gene (20% of the genes). The other encoded *lipopolysaccharide binding protein *(*lbp*),*immune reactive putative protease inhibitor *(*irpp-inhibitor*), *CHK1 checkpoint homolog*, and other putative genes, respectively (Fig. 1B).

### Identification of genes differentially expressed during metamorphic molting

**Figure 2:**
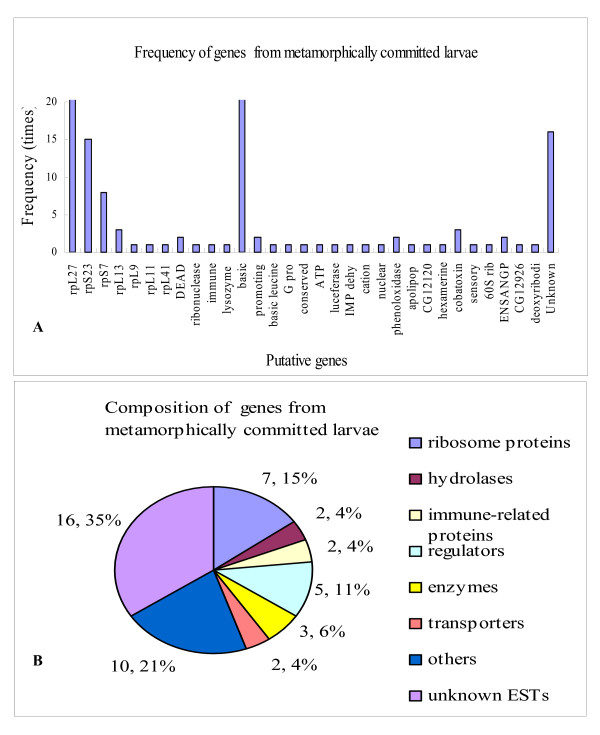
Genes identified in metamorphically committed larvae. A) Gene frequency. B) Gene transcription composition (number, percentage).

Two hundreds cDNA fragments were sequenced from SSHs between tester cDNAs of metamorphically committed larvae (6th-72, 96 and 120 h) and driver cDNAs of feeding 5th instar larvae (5th-24 h). By BLAST search, 31 putative genes with similarity to annotated Genbank genes, and 16 unknown ESTs were identified (Table 2). The most frequently appearing genes were ribosome protein encoding genes, including *rpL27*, *S23 *and *S7*. Another frequently occurring gene was the basic *juvenile hormone-suppressible protein *(*BJHSP*), which was not detected in 5th molting or 5th feeding larvae by SSH. Other genes identified were specifically detected only once or twice in this SSH (Fig. 2A).

These genes were classified into eight groups, as described above: ribosome proteins, hydrolases, immune-related genes, regulators, enzymes, transporters, others and unknown ESTs. A large number of unknown ESTs were common to tissues from the metamorphic molt (Fig. 2B). In addition, some important genes such as *BJHSP*, *DEAD-box RNA helicases*, *basic leucine zipper *(*bzip*), and *guanine nucleotide binding protein gamma subunit *(*G-protein-γ*) were detected at these stage.

### Identification of genes differentially expressed in feeding 5th instar larvae

**Table 2:** Identification of putative genes from SSH of tester cDNA from metamorphically committed larvae and driver cDNA from feeding 5th instar larvae

**Classification**	**No**	**Tissue sources**	**Similar genes in GenBank**	**E-value**	**Functions of the genes**
Ribosome proteins	1	epidermis	ribosome protein S23 [*D.melanogaster*]	4e-06	see Table 1
	2	epidermis Fat body midgut head	ribosome protein L27 *[S. frugiperda]*	4e-54	see Table 1
	3	fat body head	ribosome protein S7 [*B.mori*]	7e-24	see Table 1
	4	epidermis midgut head	ribosome protein L13 [*Helicoverpa zea*]	2e-24	see Table 1
	5	head	ribosome protein L9 [*B. mori*]	2e-26	compose the large ribosomal subunit
	6	head	ribosome protein L11 [*B. mori*]	5e-22	large ribosomal subunit protein
	7	head	ribosomal protein L41 [*S. frugiperda*] (BLAST by nucleotide acids)	1e-23	mitochondrial ribosomal protein
Hydrolases	8	fat body	DEAD box RNA helicase [*C. fumiferana*]	1e-25	disrupt RNA-protein interactions
	9	midgut	ribonuclease [*Ceratitis capitata*]	8e-31	degradation of RNA
Immune-related proteins	10	midgut	immune inducible protein [*B. mori*]	3e-11	immune-response
	11	midgut	lysozyme [*Heliothis virescens*]	1e-17	immune
Regulator	12	epidermis	basic juvenile hormone-suppressible protein 2 precursor (BJHSP2) [*S. litura*]	6e-08	metamorphosis related
	13	epidermis	promoting protein [*B. mori*]	3e-12	promoting in vitro replication of nucleopolyhedrovirus
	14	head	basic leucine zipper and W2 domains 2 [*R. norvegicus*]	0.009	transcription factor controlling molting and metamorphosis
	15	fat body	guanine nucleotide binding protein gamma subunit-like protein [*P. xylostella*]	4e-22	Signal transduction
	16	midgut	conserved hypothetical protein [Lactococcus lactis]	5e-19	-
Enzyme	17	fat body	ATP synthase subunit 6 [*Phthonandria atrilineata*]	0.15	ATP synthase
	18	midgut	luciferase [*Luciola lateralis*]	8e-13	Fatty acyl-CoA synthetase
	19	head	IMP dehydrogenase/GMP reductase [*Desulfuromonas acetoxidans *DSM 684]	0.31	re-utilization of free intracellular bases and purine nucleosides
Transporter	20	epidermis	cation channel family protein [*Tetrahymena thermophila SB210*]	6.1	Ca^2+^-permeable ion channel
	21	midgut	nuclear transport factor 2 [*Aedes aegypti*]	3e-24	targeting proteins into the nucleus
Others	22	midgut	phenoloxidase inhibitor protein [*Anopheles gambiae*]	0.006	enzyme inhibitor
	23	epidermis	apolipophorin-III precursor [*Agrius convolvuli*]	1e-12	lipid transfer and cell death
	24	midgut	CG12120-PA [Tribolium castaneum]	2e-09	-
	25	epidermis	hexamerine [*H. armigera*]	2e-04	storage protein during metamorphosis
	26	midgut	cobatoxin long form B [*S. frugiperda*]	8e-05	-
	27	midgut	sensory neuron membrane protein 2 [*M. sexta*]	4e-05	-
	28	head	60 S ribosome subunit biogenesis protein [*Theileria annulata*]	9.7	-
	29	midgut	ENSANGP00000017373 [*Apis mellifera*]	3e-10	-
	30	head	CG12926-PA [*T. castaneum*]	3e-19	-
	31	midgut	deoxyribodipyrimidine photolyase [Marinobacter aquaeolei VT8]	6.1	-
Unknown ESTs	32–47	midgut epidermis fat body head			

**Figure 3:**
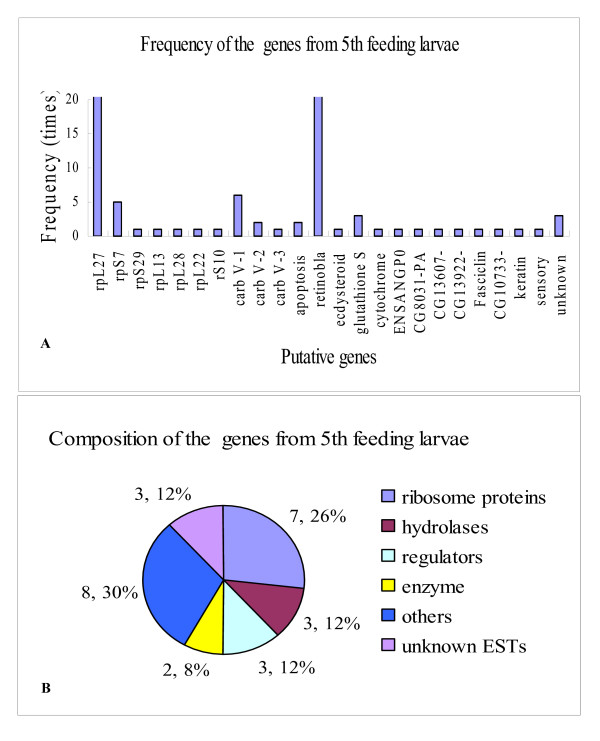
Genes identified in 5th instar feeding larvae. A) Gene frequency. B) Gene composition (number, percentage).

SSHs between feeding 5th instar larvae tester and metamorphically committed larvae (6th-72, 96 and 120 h) driver were performed to identify cDNAs enriched in feeding 5th instar larvae. In total, 230 cDNA fragments were sequenced following PCR. Of these, 23 putative genes and 3 unknown ESTs were identified (Table 3). Ribosome protein encoding genes were the most common, especially *rpL27 *that appeared very higher frequency, whereas other ribosome proteins had lower frequencies. Another high frequency gene was the gene encoding retinoblastoma-binding protein 6 isoform 2-like, which was not identified in molting 5th instar or metamorphically committed larvae by SSH. Other lower frequency genes were specifically detected in feeding 5th instar larvae (Fig. 3A).

**Table 3:** Identification of putative genes from SSH of tester cDNA from feeding 5th instar larvae and driver cDNA of metamorphically committed larvae

**Classification**	**No**	**Tissue sources**	**Similar genes in GenBank**	**E-value**	**Functions of the genes**
ribosome proteins	1	head midgut epidermis	ribosomal protein L27 [*S. frugiperda*]	9e-55	see Table 1
	2	midget	ribosomal protein L22 [*S. frugiperda*]	9e-07	see Table 1
	3	midgut	ribosomal protein S29 [*S. frugiperda*]	5e-29	apoptotic inducer
	4	head	ribosomal protein L13 [*H. zea*]	5e-43	see Table 1
	5	midgut	ribosomal protein L28 [*S. frugiperda*]	6e-38	ribosome assembly
	6	midgut	ribosomal protein S7 [*Plutella xylostella*]	7e-25	see Table 1
	7	midgut	ribosomal protein S10 [*Lonomia obliqua*]	1e-33	mitochondrial import proteins
hydrolases	8	epidermis	carboxypeptidase, vitellogenic-like-1 [*T. castaneum*]	1e-29	inflammatory protease cascade
	9	epidermis	carboxypeptidase, vitellogenic-like-2 *[T. castaneum*]	2e-16	inflammatory protease cascade
	10	epidermis	carboxypeptidase, vitellogenic-like-3 [*D. rerio*]	6e-04	inflammatory protease cascade
regulators	11	epidermis	apoptosis inhibitor [*Bos taurus*]	1e-16	candidate of molting-related protein
	12	midgut	ecdysteroid-regulated protein [*Litopenaeus vannamei*]	5e-19	molting-related protein
	13	head	retinoblastoma-binding protein 6 isoform 2 [D. rerio]	0.87	candidate of molting-related protein
enzyme	14	head fat body	glutathione S-transferase [*C. fumiferana*]	9e-09	detoxification of electrophilic compounds
	15	midgut	cytochrome P450 like_TBP [*Nicotiana tabacum*]	1e-10	^-^
others	16	epidermis	ENSANGP00000014082 [*A. gambiae*]	1e-17	-
	17	epidermis	CG8031-PA [*D. Melanogaster*]	1e-35	-
	18	midgut	CG13607-PA [*T. castaneum*]	1e-18	-
	19	midgut	CG13922-PA [*T.castaneum*]	4e-05	-
	20	midgut	fasciclin 3 CG5803-PA-isoform A [*D. melanogaster*]	8.0	-
	21	midgut	CG10733-PAisoform [*T. castaneum*]	8e-06	-
	22	fat body	keratin associated protein 7 [*Pan troglodytes*]	0.98	-
	23	epidermis	sensory box histidine kinase/response regulator	6.2	-
Unknown ESTs	24–26	midgut fat body epidermis	unknown proteins		-

Genes were classified into 6 groups, according to similarity, including ribosome proteins, hydrolases, regulators, enzymes, other genes, and unknown ESTs (Fig. 3B). Interestingly, three high frequency hydrolases genes were all *vitellogenic-like carboxypeptidase*, although their sequences were quite divergent. These three genes were detected in epidermis of feeding 5th instar larvae. The genes encoding ecdysteroid-regulated protein and a *cytochrome P450 like-TBP*-like gene were also detected from feeding 5th instar larvae. In addition, many genes with unknown function were detected.

### Analysis of SSH efficacy by dot blot hybridization

**Figure 4:**
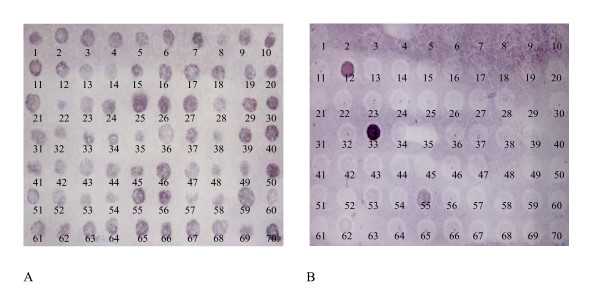
Dot blot hybridization of SSH from molting 5th instar epidermis tester and feeding 6th instar epidermis driver. A) Blots with spotted with cDNA fragments from SSH were hybridized with probes made from molting 5th instar epidermal mRNA. B) Blots hybridized with probes made from feeding 6th instar epidermis.

To analyze the efficacy of SSHs, dot blot hybridization was performed using 70 cDNA fragments that were randomly chosen from the set of differentially expressed genes resulting from SSH between molting 5th instar epidermis tester and feeding 6th instar epidermis driver. Blots were hybridized with probes made from cDNA of molting 5th instar larval epidermis. Nearly all of the 70 dots stained positively. In contrast, when blots were hybridized with probes made from cDNA of feeding 6th larval epidermis, only 3 dots were obviously positively stained. This result indicates that 95% of the cDNAs detected by SSH were differentially expressed during larval molting, while 5% of the cDNAs may not be molting-specific (Fig. 4).

### Northern blot analysis of gene expression

**Figure 5:**
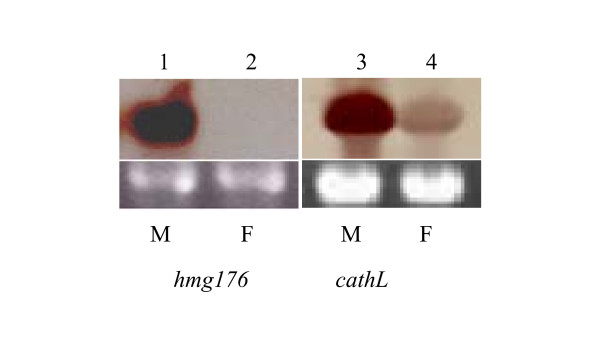
Northern Blot analysis of the expression of *hmg176 *and *cathL *in molting 5th and feeding 6th instar larvae. Lanes 1 to 4: midgut from molting 5th instar larvae, midgut from feeding 6th instar larvae, hemocytes from molting 5th instar larvae, and hemocytes from feeding 6th instar larvae. M = 5th-HCS, F = feeding.

Two genes identified by SSH were chosen for analysis by Northern blot in different tissues at various developmental stages. One of these was *hmg176*, which was identified in molting 5th instar midguts by SSH between molting 5th instar larvae tester and feeding 6th instar driver. The other gene examined was *cathL*, identified in molting 5th instar hemocytes by the same SSH. The results indicated that *hmg176 *was highly expressed in the midgut during larval molting, but was not detected in 6th-48 h larvae. Similarly, *cathL *was obviously upregulated in hemocytes of molting larvae (Fig. 5). These data suggest that some of the ESTs identified by SSHs do represent stage-specific genes.

### Semi-quantitative RT-PCR analysis of larval molting or metamorphosis genes

To further analyze larval molting and metamorphosis genes and to examine gene expression patterns in different tissues, such as head, midgut, fat body, epidermis, and hemocytes during development, 22 putatively differentially expressed genes were randomly chosen for semi-quantitative RT-PCR analysis. These included 18 genes from SSHs between molting 5th instar larva testers and feeding 6th instar larva drivers, 3 genes from SSHs between metamorphically committed larva testers and feeding 5th instar drivers, and 1 gene from SSHs between feeding 5th instar testers and metamorphically committed larva drivers. The *beta-actin *gene was used as a positive control.

Of these genes, 8 genes were differentially detectable during larval molting. Some genes were known larval molting genes, such as *carbA1*, *irpp-inhibitor*, *hmg176*, *carbA2 *and a *tumor necrosis factor receptor *(*TNFR*)-like gene. Interestingly, these genes exhibited distinct tissue-specific expression patterns as well. For instance, *carbA1 *was mainly detected in molting larval fat bodies, epidermis and hemocytes, the *irpp-inhibitor *was mainly detected in molting larval fat bodies and epidermis, *hmg176 *was mainly expressed in molting larval midguts, *carbA2 *was mainly detected in molting larval epidermis; and the *TNFR*-like gene was mainly detected in molting larval heads, midguts and fat bodies. An unknown EST was detectable at higher levels in all 5 tissues of molting larvae than in feeding larvae. *CathL *was detected at higher levels in molting larval hemocytes than feeding larval hemocytes, consistent with Northern blot analysis. The *tPA *gene was detected at higher levels in molting larval fat bodies compared to feeding larval fat bodies.

Some genes exhibited differential expression in different tissues during larval molting. For example, the *Na-K-ATPase*-like gene was detected at higher levels in the epidermis, but not in the head, midgut, or hemocytes of molting larvae. *Titin *appeared to be expressed at higher levels in the midgut, but not in other tissues, of molting larvae. *Trypsinogen Y precursor *and *lipase *only exhibited increased expression in molting larval fat bodies compared with other detected tissues.

**Figure 6:**
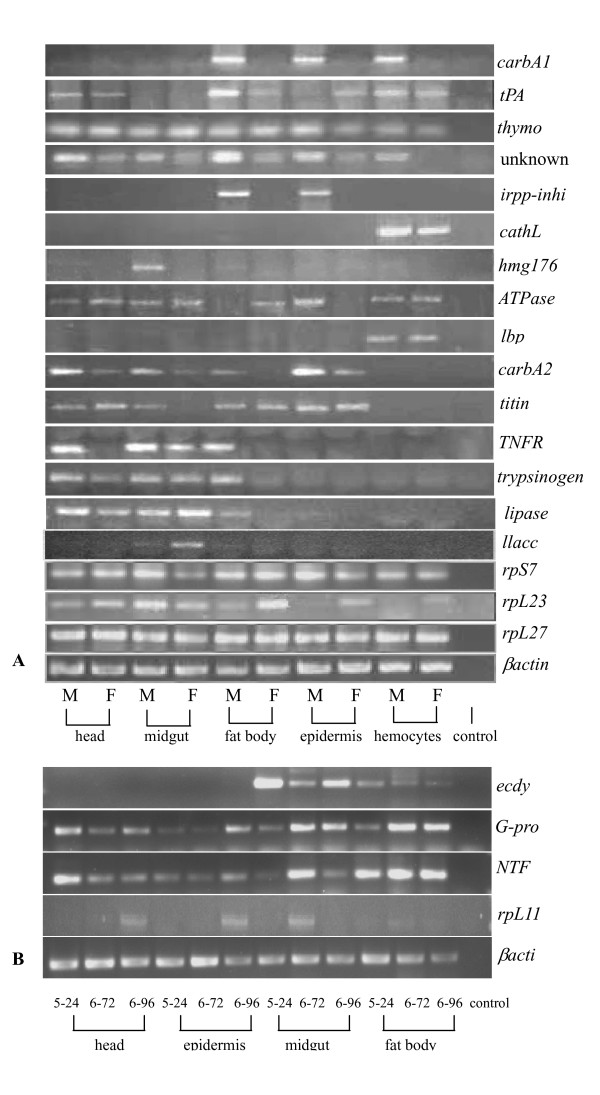
Semi-quantitative RT-PCR analysis of gene expression in tissues from different developmental stages. Panel A) Gene expression during larval molting and feeding, M = molting 5th instar larvae (5th-HCS), F = feeding 6th instar larvae (6th-48 h). Panel B) gene expression during metamorphosis. 5th-24 = 5th instar larvae 24 h after ecdysis, 6th-72 h = 72 h after ecdysis (wandering-0 d, metamorphically committed larvae); 6th-96 h, 96 h after ecdysis (wandering-1 d, metamorphically committed larvae).

Some genes did not show an obvious relationship to larval molting. For example, *lbp *was not differentially detected between larval molting and feeding by semi-quantitative RT-PCR, although it was detected specifically in hemocytes. Expression of the *thymosin beta homologue-like *gene, *rpL27 *and *S7 *were detected at almost constant levels in molting and feeding larval tissues. *RpL23 *and the hypothetical protein gene (*llacc*) appeared higher expression during feeding in some tissues (Fig. 6A).

Three genes were differentially detected by semi-quantitative RT-PCR in a tissue-specific manner during metamorphosis. These were the genes encoding the *gamma subunit of guanine nucleotide binding protein *(*G-protein-γ*), *nuclear transport factor-2 *(*NTF-2*) and *rpL11*. The *G-protein-γ *was detected at higher levels in the epidermis, midgut and fat body of the metamorphically committed larvae in 6th-72 or 6th-96 h compared with the tissues from feeding 5th larvae. The *NTF-2 *was also detected at higher levels in the midgut of metamorphically committed larvae. The *rpL11 *was detected at higher levels during metamorphosis and in all 4 examined tissues compared with the tissues from feeding 5th larvae. In contrast, the *ecdysteroid-regulated *gene was upregulated in the tissues from feeding larvae, which is consistent with the fact that it was detected in feeding 5th instar larvae by SSH (Fig. 6B).

## Discussion

We identified 100 ESTs, including 73 putative genes that were similar to genes in Genbank, and 27 unknown ESTs by SSH of larval tissues. Most similar genes had E-values below 0.01, while less similar genes had E-values greater than 0.01 (Tables 1,2,3). Eleven of the putative genes were differentially detectable during larval molting or metamorphosis via semi-quantitative RT-PCR, including regulatory genes expressed during molt and metamorphosis such as NTF-2, G-proteins, and the downstream genes that may play a direct role in the molting and metamorphosis such as carbA2 and cathL. Expression levels of these genes clearly correlated with larval molting or metamorphosis in a largely tissue-specific or developmental stage-specific manner by semi-quantitative RT-PCR analysis. Importantly some of these genes have been reported to play a role in larval molting or metamorphosis. For examples, *carbA2 *has been reported to be a molting gene in *Bombyx*, and is expressed during molting in the epithelial tissues [16]. The CarbA2 protein has been suggested to degrade old cuticle during molting and to contribute to recycling of the amino acids in this tissue [16]. *CathL*, which encodes a cysteine proteinase, is involved in larval molting and cuticle and eggshell remodeling in *Brugia pahangi *[17]. Other genes identified in this study have not been previously known to play a role in molting or metamorphosis. Their predicted function, based on similarity, however, supports the notion that these genes participate in regulation of larval molting or metamorphosis. For instance, nuclear transport factor-2 (NTF-2) targets proteins to the nucleus in yeast and mammalian cells in culture [18]. The gamma subunit of guanine nucleotide binding protein (G-protein-γ) is an essential component of one of the most prevalent signaling systems in mammalian cells. The fact that *NTF-2 *and *G-protein-γ *are upregulated during metamorphosis suggests that they participate in metamorphosis.

Some genes identified in this study, based on functions of similar genes, probably participate in larval molting or metamorphosis, although they have not yet been demonstrated to be upregulated in larval molting or metamorphosis in this study. For instance, the basic-leucine zipper gene, controls molting and metamorphosis and is likely to be involved in bZIP signaling pathways in *Drosophila *[19]. The basic juvenile hormone-suppressible protein 2 (BJHSP2), a juvenile hormone-sensitive protein, expresses and accumulates in the last instar larvae of *Trichoplusia ni *[20]. The DEAD box RNA helicase and leucine-rich repeat genes are important in molting *C. elegans *[6]. The Chk1 checkpoint protein was reported to monitor the state of DNA and can delay or arrest the cell cycle at multiple points [21]. The ribonuclease selectively attacks malignant cells, triggering an apoptotic response and inhibiting protein synthesis [22]. These putative genes represent attractive targets for further study into their functions in larval molting and metamorphosis.

Ribosomal proteins have been reported to participate in various cellular processes besides protein biosynthesis. They not only act as components of the translation apparatus, but also regulate cell proliferation and apoptosis [23]. For example, rpL11 associates with and inhibits the transcriptional activity of peroxisome proliferator-activated receptor-alpha [24]. RpL23 regulates p53-MDM2 feedback and the HDM2-p53 pathway [25,26]. Mitochondrial ribosomal protein L41 plays an important role in p53-induced mitochondrion-dependent apoptosis by enhancing p53 stability and contributing to p53-induced apoptosis [27,28]. RpS29 induces apoptosis in H520 cells [29,30]. RpS10 participates in both transcription and translation [31]. We detected these ribosome proteins, and they appear to be highly conserved. Among these, *rpL11 *was differentially expressed during metamorphosis. Whether or not other ribosome proteins participate in larval molting and metamorphosis need further study.

Interestingly, several putative immune-related genes were identified in molting larvae in this study. For instance, a lysozyme was identified in metamorphically committed larvae. Interestingly, a lysozyme has also been reported to be upregulated during larval metamorphosis in *M. sexta*, and has been proposed to provide protection from bacterial infection [32]. Other immune-related genes including the immune inducible protein and IRPP-inhibitor might play a similar role. These observations provide evidence that some immune-related genes are deferentially expressed during larval molting or metamorphosis.

We also detected an ecdysteroid-regulated protein-like gene encoding an ML domain (MD-2-related lipid-recognition domain) in feeding 5th instar larvae. Single ML domain proteins are predicted to form a beta-rich fold containing multiple strands and to mediate diverse biological functions through interaction with specific lipids [33]. The function of this gene in larval development is unknown. In addition, three kinds of carboxypeptidase vitellogenic-like genes (*cpvl*) were detected in the epidermis of feeding 5th instar larvae. Human *cpvl *is upregulated during the maturation of monocytes (MO) to macrophages, and is involved in antigen processing, the secretory pathway and/or in actin remodeling and lamellipodium formation [34,35]. Because these genes were detected in feeding 5th instar feeding larvae, they may play important roles in larval growth and development.

We examined the efficacy of SSH by dot blot, Northern blot and RT-PCR. Dot blot analysis of cDNAs from the epidermis of molting 5th instar tester and feeding 6th instar driver suggested the efficacy was 95%. Northern blot analysis indicated that some were differentially expressed during larval molting. However, RT-PCR analysis suggested that only 11 of the 22 examined genes were differentially expressed during larval molting or metamorphosis, and 4 genes, including the *Na-K-ATPase-like*, *titin*, *trypsinogen Y precursor*, and *lipase*, were only upregulated in some tissues during larval molting. Therefore, the efficacy of SSH was not as high as indicated by the dot blot analysis. This discrepancy might be due to limitation in sensitivity of the dot blot.

Intriguingly, several known genes critical for larval molting and metamorphosis in other species were not identified in this study. One possible explanation might be because that transcripts of these genes, most of them are transcription factors, are extremely rare, or they are expressed for a very short time. Another possible explanation is that too few cDNAs were sequenced.

Finally, certain genes, including *rpL27*, S7, and *L13 *were detected at higher levels in all SSHs. This might be due to incomplete subtraction of the extremely highly expressed housekeeping genes or to excessive cycles of PCR amplification. Sampling mRNA at correct developmental stages of larval molting and metamorphosis, increasing the amount of driver cDNA used, reducing the number of PCR cycles, and sequencing more cDNAs might improve the efficacy of SSH in future studies.

Of the 11 genes upregulated during larval molting or metamorphosis, 8 were differentially detected during larval molting (HCS), including *carbA1*, *irpp-inhibitor*, *hmg176*, *carbA2 *and a *tumor necrosis factor receptor *(*TNFR*)-like gene, and 3 were differentially detected from metamorphically committed larvae, including the *gamma subunit of guanine nucleotide binding protein *(*G-protein-γ*), *nuclear transport factor-2 *(*NTF-2*) and *rpL11*. According to studies in *Manduca*, 20E levels peak during larval molting several hours before HCS and then decrease to lower levels while JH levels peak at larval ecdysis. 20E and JH again peak together at pupal commitment stage (wandering stages). After pupation, 20E peak again during pupal development [4]. *Helicoverpa armigera *belongs to Lepidoptera: Noctuidae and has similar 20E titer to *M. sexta*, which reaches peak levels during 5th and 6th larval molts and decreases to lower level during ecdysis [36]. The 11 differentially expressed genes detected in this study are likely regulated by the puff of 20E, which present us new gene candidates for further study the functions and regulatory expression of these genes during larval molting or metamorphosis.

## Conclusion

From the 100 ESTs identified from SSH of various tissues of *H. armigera*, we identified 11 that are differentially upregulated during larval molting or metamorphosis. In addition, we present several other candidates for further studies of larval molting or metamorphosis. These studies will extend our knowledge the regulatory mechanisms of holometabolous insect larval molting and the metamorphosis cascade.

## Methods

### Insects

The cotton bollworms, *Helicoverpa armigera *(Lepidoptera: Noctuidae), were reared on food composed mainly of wheat, soybean, vitamins, and inorganic salts, and maintained in a light:dark cycle of 14:10 h at 27°C. Developmental stages were defined as follows. Fifth-0 h larvae: immediately after ecdysis with white head capsule (5th-0 h, WH). 5th instar larvae normally eat for 36~ 40 h and then head capsule slippage (HCS) occurs. HCS lasts for about 12 h, during which larvae considered 5th instar-molting larvae (5th-molting, or 5th-HCS). Next, larvae shed their cuticle in a few minutes and enter the 6th instar (6th-0 h, WH). Sixth instar larvae eat for 48 h, then purge the gut and turn red, and begin wandering at 72 h (6th-72 h, W-0 d). Larvae continue wandering until 96 h (6th-96 h, W-1 d), then contract their legs and stop wandering at 120 h (6th-120 h, PP), and pupation begins at 140 h (P-0 h/d). Pupae are designated P-1 d, P-2 d, and so on. Pupae develop for 9 ~ 10 d, depending on sex, until eclosion. According to studies in *Manduca*, 20E levels peak several hours before HCS and then begin to decrease. JH levels peak at larval ecdysis. 20E and JH again peak together at wandering and prepupal stages [4]. We prepared all samples for this study according to these developmental markers in individuals of equal body size.

### Isolation of mRNAs from issues and synthesis of cDNA

Messenger RNA was isolated from epidermis, midguts, fat bodies, hemocytes, and heads at four developmental stages using Micro mRNA Isolation kit (Amersham, Uppsala, Sweden) following the manufacturer's protocol. Developmental stages included the 5th to 6th instar transition, the feeding 6th instar stage (6th-48h), metamorphically committed larvae (6th-72, 96, and 120 h), and feeding 5th instar larvae (5th-24 h). First strand cDNAs were synthesized using SMART subtract cDNA construction kit from Clontech (BD Bioscience Clontech, Mountain View, USA) from 2 μg of mRNA with the following primers: 5' SMART CDS Primer II A Oligonucleotide and 3' SMART CDS Primer II A (5'-AAGCAGTGGTATCAACGCAGAGTACGCGGG-3', 5'-AAGCAGTGGTATCAACGCAGAGTACT_(30)_N_-1_N_-3_-3', N_-1 = _ACG; N= ATCG). Second strands were synthesized from first strand cDNAs using PCR Primer II (5'-AAGCAGTGGTATCAACGCAGAGT-3').

### Suppression Subtractive Hybridization (SSH)

Three kinds of SSH were performed, including tester cDNAs from five tissues from molting 5th instar larvae and driver cDNAs from five tissues from feeding 6th instar larvae, tester cDNAs from five tissues from metamorphically committed larvae and driver cDNAs from five tissues from feeding 5th instar, and tester cDNAs from five tissues of feeding 5th instar larvae and driver cDNAs from five tissues from metamorphically committed larvae.

cDNAs from testers and drivers were first cut into fragments by *Rsa*I digestion. Each cDNA from the testers was then separated into two portions and adapters 1 and 2R were each added to one of the two parts.

5'-CTAATACGACTCACTATAGGGCTCGAGCGGCCGCCCGGGCAGGT-3' adapter 1

3'-GGCCCGTCCA-5'

5'-CTAATACGACTCACTATAGGGCAGCGTGGTCGCGGCCGAGGT-3' adaptor 2R

3'-GCCGGCTCCA-5'

Testers were then separately hybridized with excess drivers in a ratio of tester:driver 1:30 for the first hybridization, and followed a second hybridization by adding 1 μl driver mixture (1 μl hybridization buffer, 1 μl driver and 2 μl water) to the first hybridization solution according to the Clontech PCR-Select cDNA SUBTRACTION kit protocol (BD Biosciences (BD Bioscience Clontech, Mountain View, USA). PCR amplification was then performed twice with PCR primer 1 (5'-CTAATACGACTCACTATAGGGC-3') and nested PCR primers 1/primer 2R (5'-TCGAGCGGCCGCCCGGGCAGGT-3'/5'-AGCGTGGTCGCGGCCGAGGT-3'), to amplify differentially expressed genes.

### Cloning and identification of DNA fragments from SSH

PCR products were purified and cloned into the pGEM T-Easy vector (Promega Company (Biosciences, Madison, WI, United States) and transformed into *Escherichia coli *DH5α. Insert sizes were screened by PCR and were sequenced. A similarity search using each DNA fragment as a query was performed using the Basic Local Alignment Search Tool (BLASTX) [37] to identify the genes.

### Preparation of digoxigenin-labeled anti-sense RNA probes

Target genes were inserted into the pGEM-T Easy plasmid. Recombinant plasmids were linearized, depending on the insertion direction of the target gene, and were used as templates for *in vitro *transcription of anti-sense dig-labelled probe using an RNA Labeling kit was used to synthesize the probe and the procedures were performed according to the manufacturer's suggestions (Boehringer Mannheim, Mannheim, Germany). The length of the target genes was about 500 bp.

### Preparation of digoxigenin-labeled DNA probes

Complementary DNAs (cDNA) synthesized from mRNAs isolated from 5th and 6th instar epidermis were used as templates for synthesizing dig-labeled DNA probes. The probes were synthesized by random primed PCR using the Dig High Prime DNA Labeling and Detection Starter Kit (Boehringer Mannheim, Mannheim, Germany).

### Dot blot hybridization

PCR products over 300 bp obtained by SSH were cloned into pGEM T-Easy plasmid and transformed into DH5α. Plasmids were amplified by PCR and the PCR products were quantified by agarose gel electrophoresis to choose candidates for dot blot hybridization. After denaturing for 10 min at 100°C, chilling on ice, and 2 μl of each PCR product was then dotted onto a positively-charged nylon membrane. After cross linking for 10 min under ultraviolet light, blots were prehybridized, hybridized, washed, and developed color. Prehybridization and hybridization were performed at 40°C. The probe was denatured at 100°C for 5 min before use. The wash conditions were: 2 × SSC + 0.1% SDS for 2 × 5 min at room temperature, followed by 0.1 × SSC + 0.1% SDS for 2 × 15 min at 68°C. Color development was achieved using the NBT/BCIP (nitroblue tetrazolium chloride/5-bromo-4-chloro-3-indolyl phosphate) solution (45 μl NBT 50 mg/ml in 70% v/v dimethylformamide, plus 35 μl BCIP 50 mg/ml in 100% dimethylformamide).

### Northern blot

Approximately 10 μg of total RNA was denatured and electrophoresed in a 5% formaldehyde-containing agarose gel. After electrophoresis, the RNA was transferred onto a nylon membrane. Target mRNA was prehybridized for 2 h and hybridized with dig-labeled antisense RNA probe (100 ng/ml final concentration) in Northern hybridization buffer, at 68°C overnight. After the stringency wash at 68°C 2 × SSC + 0.1% SDS for 2 × 5 min at room temperature, followed by 0.1 × SSC + 0.1% SDS for 2 × 15 min at 68°C, anti-dig-Phosphatase AP antibody (1:10000 diluted in buffer 2, 100 mM maleic acid, 150 mM NaCl; pH 7.5, 1% blocking reagent) was used to detect the probe at 37°C. NBT and BCIP were used to visualize the signals as description above. All steps were carried out as recommended by the manufacturer (Roche).

### Semi-quantitative reverse transcription-PCR (Semi-quantitative RT-PCR)

Total RNA was isolated from epidermis, midguts, fat bodies, hemocytes and heads of molting 5th instar and feeding 6th instar larvae. Complementary DNAs (cDNA) were then synthesized. The beta-actin gene was used as a cDNA quantity control. PCR conditions were as follows: 94°C for 2.5 min, and then 23 cycles of 94°C for 45 s, 53°C for 1 min and 72°C for 1 min, followed by a single cycle at 72°C for 10 min. The primers used are listed in Table 4.

**Table 4:** List of primers for semi-quantitative RT-PCR

Number	genes	primers
1	carboxypeptidase A1	CarbA1F2 5'-gcattatcttgattcgggtc-3' CarbA1RT 5'-catcttgcccacagtttc-3'
2	cathepsin L	CathLF1 5'-gcggaatacggacaatacac-3' CathLR2 5'-tagtgattagcgtggtcgcg-3'
3	lipopolysaccharide binding protein	LipoF2 5'-acttcgctgcagtcttcaag-3' LipoR2 5'-cgtcgtgcctgtgctattgc-3'
4	immune reactive putative protease inhibitor	InhibiF1 5'-tttgagttgcgttgtggtgg-3' InhibiR2 5'-tcaccaacaggtatgcattc-3'
5	lipase	LipaseF2 5'-aggatttgggcgatttcatc-3' LipaseR2 5'-ttggtcaagcagcctggttg-3'
6	trypsinogen Y precursor	TrypF2 5'-ggttggggaagtcgaaactc-3' TrypR2 5'-ctcaggaaaagctctaacag-3'
7	conserved hypothetical protein [Lactococcus lactis]	LactF2 5'-ttagttaccaagattggctc-3' LactR2 5'-gaatgtcaaaccacttgtgc-3'
8	titin-like protein	TitinF1 5'-gaggaaatcgttgacgacac-3' TitinR2 5'-tttctaccttcggacctacc-3'
9	Na-K-ATPase	ATPaseF2 5'-atactgaggacctgccttcc-3' ATPaseR2 5'-actggcccgatgttctccac-3'
10	hmg176	HMG176F 5'-atgaaaagtttccttgtcatc-3' HMG176R 5'-cttaatctaaccaagaaaccac-3'
11	carboxypeptidase A2	CarbA2F2 5'-ccagaatctaaagcaattgc-3' CarbA25-P 5'-aaagccttaacggccagtgtc-3'
12	tissue plasminogen activator	tPAF2 5'-tggtatcaacgcagagtagc-3' tPAR3 5'-atgagagtatttgcccgtgg-3'
13	thymosin beta homologue	ThymoF1 5'-ggaacccaaaccggttcaag-3' ThymoR1 5'-attcagtcttgtcagcgacc-3'
14	TNF receptor homolog 3	TNFF1 5'-aagtttccttgtcatcgccc-3' TNFR1 5'-ctactagaagctcctgtgtc-3'
15	unknown EST	UNF1 5'-aacatgtcccagcggctaac-3' UNR1 5'-tcaacctgtcagcgtgacag-3'
16	ribosome protein S23	S23F 5'-ggtacgcgggattgaagagaacg-3' S23R 5'-caacacagctttgcagcagcagg-3'
17	ribosome protein L27	L27F 5'-cctcagacaagccctacggac-3' L27R 5'-ctcttgtatctctcctcgaag-3'
18	ribosome protein S7	S7F 5'-catccggatcaagctcgacgg-3' S7R 5'-ttcctaagtttacaagtaggg-3'
19	ribosome protein L11	L11F 5'-catctgtgtgggagagtctggtg-3' L11R 5' – gtgttgtcagtctcactcgctgc-3'
20	G-protein-γ	Gproγ 5'-atggatatgatggtatcaacg-3' Gproγ 5'-ttaaagaacagtgcaggaact-3'
21	NTF-2	NTF2F 5'-atggcgctcaatccacaatac-3' NTF2R 5'-ctttgaaggagtacagttgca-3'
22	ecdysteroid-regulated gene	EcdyrF 5'-ctgcaggatgccgtcatac-3' EcdyrR 5'-ctaggcgcgcggaggagcgat-3'
23	beta-actin	ActinF 5'-agtagccgccctggttgtagac-3' ActinR 5'-ttctccatgtcgtcccagt-3'

## Authors' contributions

Du-Juan Dong constructed the SSH library and analyzed the DNA sequences. Hong-Juan He constructed the SSH library and analyzed the DNA sequences. Lian-Qin Chai carried out RT-PCR and Northern blot. Xiao-Juan Jiang constructed the SSH library and did Dot blot hybridization and Northern bolt. Jin-Xing Wang participated in its design and coordination. Xiao-Fan Zhao participated in the design of the study and drafted the manuscript. All authors read and approved the final manuscript.

## Accession numbers

Accession numbers of the putative genes and unknown expression sequence tags (ESTs) in this study are: [GenBank: DQ875214–DQ875283 and EE332468–EE332497].
